# Karyotypic evolution of the *Medicago* complex: *sativa-caerulea-falcata* inferred from comparative cytogenetic analysis

**DOI:** 10.1186/s12862-017-0951-x

**Published:** 2017-04-21

**Authors:** Feng Yu, Haiqing Wang, Yanyan Zhao, Ruijuan Liu, Quanwen Dou, Jiangli Dong, Tao Wang

**Affiliations:** 1 Key Laboratory of Adaptation and Evolution of Plateau Biota, Northwest Institute of Plateau Biology, Chinese Academy of Sciences, Xining, 810008 China; 2 University of Chinese Academy of Sciences, Beijing, 100049 China; 3 State Key Laboratory of Agrobiotechnology, College of Biological Sciences, China Agricultural University, Beijing, 100193 China

**Keywords:** *Medicago sativa*, *M. sativa* ssp. *caerulea*, *M. sativa* ssp. *falcata*, Repetitive sequences, FISH, Chromosome evolution, Diploidization

## Abstract

**Background:**

Polyploidy plays an important role in the adaptation and speciation of plants. The alteration of karyotype is a significant event during polyploidy formation. The *Medicago sativa* complex includes both diploid (2n = 2× = 16) and tetraploid (2n = 2× = 32) subspecies. The tetraploid *M.* ssp. *sativa* was regarded as having a simple autopolyploid origin from diploid ssp. *caerulea*, whereas the autopolyploid origin of tetraploid ssp. *falcata* from diploid form ssp. *falcata* is still in doubt. In this study, detailed comparative cytogenetic analysis between diploid to tetraploid species, as well as genomic affinity across different species in the *M. sativa* complex, were conducted based on comparative mapping of 11 repeated DNA sequences and two rDNA sequences by a fluorescence in situ hybridization (FISH) technique.

**Results:**

FISH patterns of the repeats in diploid subspecies *caerulea* were highly similar to those in tetraploid subspecies *sativa*. Distinctly different FISH patterns were first observed in diploid ssp. *falcata*, with only centromeric hybridizations using centromeric and multiple region repeats and a few subtelomeric hybridizations using subtelomeric repeats. Tetraploid subspecies *falcata* was unexpectedly found to possess a highly variable karyotype, which agreed with neither diploid ssp. *falcata* nor ssp. *sativa*. Reconstruction of chromosome-doubling process of diploid ssp. *caerulea* showed that chromosome changes have occurred during polyploidization process.

**Conclusions:**

The comparative cytogenetic results provide reliable evidence that diploid subspecies *caerulea* is the direct progenitor of tetraploid subspecies *sativa*. And autotetraploid ssp. *sativa* has been suggested to undergo a partial diploidization by the progressive accumulation of chromosome structural rearrangements during evolution. However, the tetraploid subspecies *falcata* is far from a simple autopolyploid from diploid subspecies *falcata* although no obvious morphological change was observed between these two subspecies.

**Electronic supplementary material:**

The online version of this article (doi:10.1186/s12862-017-0951-x) contains supplementary material, which is available to authorized users.

## Background

Polyploidy is very common in plant evolution. It plays an important role in adaptation and speciation of plants [1]. According to different chromosome set origins, polyploidy is generally classified into autopolyploid and allopolyploid [2]. The structural changes of genome including chromosome fusions, chromosome number reduction, and a variety of chromosome rearrangements were a significance event during polyploidy formation [3]. It has been illustrated in many allopolyploid species, such as *Nicotiana* [3, 4], *Tragopogon* [5], *Gossypium* [6, 7] and *Brassica* [8, 9]. In *Nicotiana*, intergenomic translocations have been detected in natural *N. tabacum* genotypes and this translocation was considered to be significant in tobacco fertility [3]. Compared with in allopolyploid, structural changes were more difficult to be discovered due to homologous genomes were duplicated in autopolyploid. However, chromosomal rearrangements were reported in induced autotetraploid *Lathyrus sativus* [10] and *Arabidopsis thaliana* [11].

The *Medicago sativa* complex includes both diploid (2n = 2× = 16) and tetraploid (2n = 2× = 32) subspecies [12]. Tetraploid subspecies *M. sativa* ssp*. sativa* L., an important world forage legume, and diploid subspecies *M. sativa* ssp. *caerulea* (Less. ex Ledeb.) Schmalh. have a similar morphology with violet flowers and coiled pods [13, 14]. Subspecies *M. sativa* ssp. *falcata* (L.) Arcang. comprises both diploid and tetraploid forms which differ morphologically from the previous two taxa by having conspicuous yellow flowers and straight to sickle-shaped pods [13, 14]. With the similar ploidy level, they intercross easily and produce viable hybrids [15]. Tetraploid ssp. *sativa* and ssp. *falcata* have been considered to be autotetraploidy due to appearance of quadrivalents at meiosis and tetrasomic inheritance [16–18]. Two diploid taxa ssp. *caerulea* and ssp. *falcata* in the complex were hypothesized to be the direct progenitor of tetraploid ssp. *sativa* and ssp. *falcata*, respectively [13, 14]. However, recent molecular evidence of chloroplast suggested *M. prostrata* may have introgression into the tetraploid ssp. *falcata* in past. Therefore, the *Medicago* complex is an interesting model for polyploidy evolutionary study especially for autopolyploid [19].

Heterochromatin distributions of diploid ssp. *falcata*, ssp. *caerulea* and tetraploid ssp. *sativa* have been analyzed by C-banding and N-banding techniques [20–24]. Comparing results showed that diploid ssp. *caerulea* had similar heterochromatin distribution with tetraploid ssp. *sativa*: constitutive heterochromatic was distributed mainly around the centromeres, telomere and interstitial region of short arms of the chromosomes and partly presented at the interstitial region of long arms of chromosomes [20–24]. On the contrary, there were few heterochromatic distributions on the telomere and interstitial region in diploid ssp. *falcata* except centromere regions [20, 21, 25]. Bauchan and Hossain’s unpublished data mentioned that there were a larger number of C-bands in tetraploid ssp. *falcata* than that had been discovered in diploid ssp. *falcata* [12].

Compared with the traditional banding techniques, fluorescence in situ hybridization (FISH), a valuable molecular cytogenetic tool, can display the molecular information on the chromosome more directly, more accurately, and more stably [26, 27]. It has been widely applied to the study of plant genomic organization, chromosome identification, and species evolution by physical mapping repetitive genes or other sequences directly onto chromosomes [27–32]. In our previous study [33], 11 tandemly repetitive sequences (nine of which were novel) were isolated from a Cot-1 library in alfalfa and a FISH-based molecular cytogenetic karyotype was well developed for tetraploid ssp. *sativa*. In this study, we present an in-depth comparative molecular cytogenetic analysis between diploid and tetraploid subspecies in *Medicago sativa* complex using repetitive sequences and FISH. Chromosome changes will be described in detail in evolution process of autotetraploidy ssp. *sativa*. The relationship of tetraploid and diploid ssp. *falcata* will be discussed.

## Methods

### Plant materials

Four diploid ssp. *caerulea*, four diploid ssp. *falcata*, and six tetraploid ssp. *falcata* samples were used as materials in this study. Accessions beginning with ‘PI’ were obtained from the National Plant Germplasm System (NPGS) of the United States Department of Agriculture (USDA). Two tetraploid ssp. *falcata* accessions, XiaNH-072X-824 and Lizj0944, were acquired from the China Germplasm Bank of Wild Species. Accession 2–6 was collected from a wild population in Xinjiang, China. A list of materials with ploidy levels and origins is given in Table 1.

**Table 1:** Materials used in this study

Subspecies	Ploidy	Identification No.	Origin
*M. sativa* ssp. *caerulea*	2×	PI 464715	Turkey, Kars
	2×	PI 212798	Iran
	2×	PI 577551	Canada, Manitoba
	2×	PI 577548	Russia
*M. sativa* ssp. *falcata*	2×	PI 631808	Russia
	2×	PI 502447	Russia
	2×	PI 631813	Russia
	2×	PI 234815	Switzerland
*M. sativa* ssp. *falcata*	4×	PI 634023	Kazakhstan
	4×	PI 634118	Kazakhstan
	4×	PI 634117	Kazakhstan
	4×	XiaNH-072X-824	China
	4×	Lizj0944	China
	4×	2–6	China

### Chromosome preparation

Root tips with a length of 1–2 cm were harvested from germinated seeds or growing plants and pretreated in ice-cold water at 4 °C for 20–24 h. Root tips were then fixed in ethanol:glacial acetic acid (3:1, *v*/v) for 4 h at room temperature. Each root tip was squashed in a drop of 45% acetic acid. Finally, the slides were stored at −80 °C before use.

### Probe preparation

Eleven tandemly repetitive DNA sequences developed in alfalfa by Yu et al. [33] were used in this study. Five of the sequences (*Ms*CR-1, *Ms*CR-2, *Ms*CR-3, *Ms*CR-4, and *Ms*CR-5) were centromeric or pericentromeric, three (*Ms*TR-1, clone 65, and clone 74) were subtelomeric, and three (E180, clone 68, and clone 87) produced multiple hybridization signals in alfalfa chromosomes [33]. We also used two rDNA regions, 5S and 18S–26S rDNA, as probes. The 5S rDNA sequence was amplified by polymerase chain reaction (PCR) using genomic DNA of alfalfa as described by Fukui et al. [34]. The plasmid pWrrn, which included fragments of wheat 18S–26S rDNA, was provided by Professor Tsujimoto (Tottori University, Japan). All purified DNA products except pWrrn were labeled by the random primer labeling method with tetramethyl-rhodamine-5-dUTP (red) or fluorescein-12-dUTP (green) (Roche Diagnostics). pWrrn was labeled with tetramethyl-rhodamine-5-dUTP (red) using the nick-translation method.

### FISH and microphotometry

FISH procedure was based on Mukai’s description [35] with minor modifications. Chromosome DNA denaturation was carried out in 0.2 M NaOH in 70% ethanol at room temperature for 8 min and then dehydrated with the cold ethanol series. The probe mixture (25 ng of each labeled probe DNA, 5–10 mg of sheared salmon sperm DNA, 50% formamide, 2 × SSC, and 10% dextran sulfate) was denatured for 5 min at 95 °C and cooled on ice. Then, the denatured probe mixture was applied on dehydrated chromosome slide. The slides were incubated in a humid chamber at 37 °C overnight. After hybridization, the slides were washed in 2× SSC three times for 5 min at room temperature and briefly dried. Chromosomes were counterstained with 4′, 6-diamidino-2-phenylindole in Vectashield mounting medium (Vector Laboratories, Burlingame, CA, USA). Images were acquired with a cooled charge-coupled device camera (Photometrics CoolSNAP) under a fluorescence microscope (Leica) and were processed with the MetaVue Imaging System. Finally, images were adjusted with Adobe Photoshop 6.0 for contrast and background optimization.

## Results

### Physical mapping of repetitive sequences on mitotic chromosomes

#### In *Medicago sativa ssp. caerulea*

Physical mapping of the 11 repetitive sequences in diploid ssp. *caerulea* accession PI 464715 was conducted by FISH (Additional file 1: Figure S1a–h). Repeat sequence *Ms*CR-1, *Ms*CR-2, *Ms*CR-3, *Ms*CR-4, and *Ms*CR-5 were physically mapped on pericentromeric regions of 14, 8, 6, 10, and 9, respectively, of the 16 chromosomes of ssp. *caerulea* (Additional file 1: Figure S1a–d). Double-target FISH further revealed that *Ms*CR-3 overlapped with *Ms*CR-2, *Ms*CR-4, and *Ms*CR-5 on four, two, and three chromosomes, respectively (Additional file 1: Figure S1b–d). All three subtelomeric sequences (*Ms*TR-1, clone 65, and clone 74) were co-localized on one end of 12–13 chromosomes (Additional file 1: Figure S1e, f). At the same time, a variation of two end of one chromosome was also detected (Additional file 1: Figure S1e). Probes E180, clone 68, and clone 87 displayed hybridization signals on 16, 15, and 14 chromosomes, respectively (Additional file 1: Figure S1g, h). Double-target FISH revealed different FISH patterns between E180 and clones 68 or 87.

**Fig. 1:**
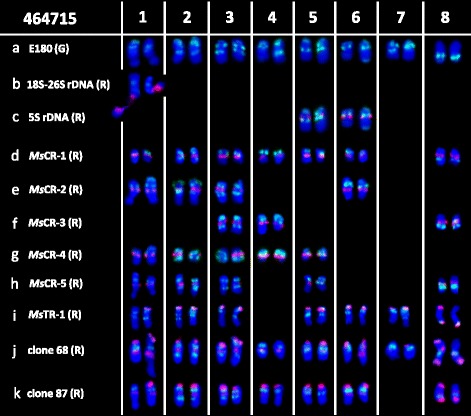
Localization of repeats on *Medicago sativa* ssp. *caerulea* PI 464715 somatic chromosomes using **a** probe E180 (*green*) in combination with **b** 18S–26S rDNA, **c** 5S rDNA, **d** *Ms*CR-1, **e** *Ms*CR-2, **f** *Ms*CR-3, **g** *Ms*CR-4, **h** *Ms*CR-5, **i** *Ms*TR-1, **j** clone 68, or **k** clone 87

The physical mapping results revealed E180 produced the greatest number of information of hybridization signals on each chromosome. Thus, double-target FISH between each repetitive sequence and E180 were carried out. Consequently, we used E180 FISH patterns, previous double-target FISH results, and chromosome arm ratios as references to allocate each sequence to a particular chromosome (Additional file 1: Figure S2a–h). The sequences were mapped as follows (Fig. 1): 18S–26S rDNA: on chromosome 1; 5S rDNA: on chromosome 5 and 6; *Ms*CR-1: on all chromosome except 7; *Ms*CR-2: on chromosome 1, 2, 3, and 6; *Ms*CR-3: on chromosome 3, 4, and 8; *Ms*CR-4: on chromosome 1, 2, 4, 5, and one of chromosome 3; *Ms*CR-5: on chromosome 1, 2, 5, 8, and one of chromosome 3; *Ms*TR-1 (co-localized with clone 65 and clone 74): on chromosome 2, 3, 5, 6, 7, 8 and one of chromosome 1; clone 68: on all chromosomes except one of chromosome 7; and clone 87: on all chromosomes except chromosome 7.

To development a standard molecular karyotype among different ssp. *caerulea* accessions, three other accessions (PI 212798, PI 577551, and PI 577548) were also used in cytogenetic analysis. Because 18S–26S rDNA, 5S rDNA, E180, *Ms*CR-3, and *Ms*TR-1 repeats showed a strong ability to distinguish chromosomes according to the results of chromosome allocation, two FISH cocktails—one consisting of 18S–26S rDNA, 5S rDNA, and E180 (Additional file 1: Figure S3a–d, Fig. 3) and the other comprising E180, *Ms*CR-3, and *Ms*TR-1 (Additional file 1: Figure S3i–l and Figure S6, Fig. 3)—were applied to these four accessions by FISH. The detailed distributions are presented in Fig. 3. Although polymorphic FISH patterns were detected on a few chromosomes among accessions, a relatively conserved karyotype was still described in Fig. 6 (a).

#### In diploid *Medicago sativa ssp. falcata*

Physical mapping of the 11 repetitive sequences in diploid *M. sativa* ssp. *falcata* accession PI 631808 was also conducted using FISH (Additional file 1: Figure S1i–p). Repeat sequence *Ms*CR-1, *Ms*CR-2, *Ms*CR-3, *Ms*CR-4, and *Ms*CR-5 were physically mapped on pericentromeric regions of 16, 10, 0–1, 9, and 8 of the 16 total chromosomes, respectively (Additional file 1: Figure S1i–l). In addition, *Ms*CR-4 and *Ms*CR-5 showed an extra band on one and two chromosomes, respectively. The three subtelomeric probes (*Ms*TR-1, clone 65, and clone 74) were co-localized on only one end of one chromosome (Additional file 1: Figure S1m, n). Probe E180 was mostly localized at a single site (mainly around the centromere) of 10 to 11 chromosomes rather than the multiple sites observed on nearly all chromosomes in ssp. *caerulea*. Similarly, clones 68 and 87 were also mostly co-localized in centromeric regions on 16 chromosomes (Additional file 1: Figure S1o, p). Double-target FISH revealed that E180 overlapped with clones 68 and 87 on nine chromosomes.

**Fig. 2:**
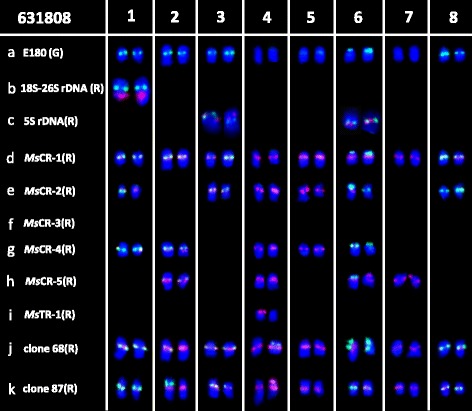
Localization of repeats on diploid *Medicago sativa* ssp. *falcata* PI 631808 somatic chromosomes using **a** probe E180 (*green*) in combination with **b** 18S–26S rDNA, **c** 5S rDNA, **d** *Ms*CR-1, **e** *Ms*CR-2, **f** *Ms*CR-3, **g** *Ms*CR-4, **h** *Ms*CR-5, **i** *Ms*TR-1, **j** clone 68, or **k** clone 87

To further characterize the chromosomes of ssp. *falcata*, hybridizations were also carried out using probe E180 and each repetitive sequence (Additional file 1: Figure S2i–p). Each sequence was allocated to a particular chromosome as follows (Fig. 2): 18S–26S rDNA: on chromosome 1; 5S rDNA: on chromosome 3 and 6; *Ms*CR-1: on all chromosome; *Ms*CR-2: on chromosome 1, 3, 4, 5, and one of chromosome 6 and 8; *Ms*CR-4: on chromosome 1, 2, 4, 5, and one of chromosome 6; *Ms*CR-5: on chromosome 2, 4, 6, and 7; *Ms*TR-1 (co-localized with clone 65 and clone 74): on one of chromosome 4; and clone 68 and clone 87: all chromosomes.

**Fig. 3:**
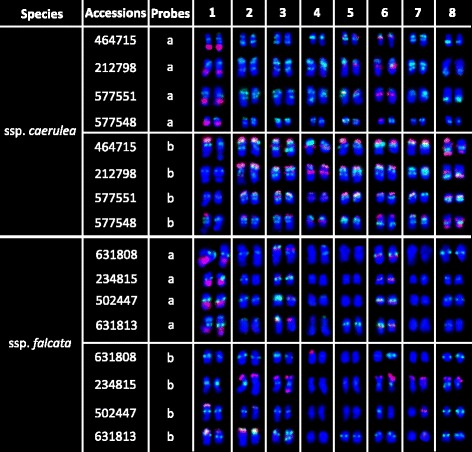
Karyotypes of four *Medicago sativa* ssp. *caerulea* accessions and four diploid *M. sativa* ssp. *falcata* accessions based on two FISH combinations. **a** Probed by E180 (*green*) combined with 18S–26S rDNA (*red*) and 5S rDNA (*red*). **b** Probed by E180 (*green*) combined with *Ms*TR-1 (*red*) and *Ms*CR-3 (red)

The same two FISH cocktails with ssp. *caerulea* were applied to diploid ssp. *falcata* PI 631808 and three other accessions (PI 234815, PI 502447, and PI 631813) to develop a standard molecular karyotype (Additional file 1: Figure S3e-h and m-p). The polymorphic distributions were presented in Fig. 3. A relatively conserved karyotype pattern was described in Fig. 6 (b).

#### In tetraploid *Medicago sativa ssp. falcata*

Physical mapping of the 11 repetitive sequences in tetraploid *M. sativa* ssp. *falcata* accession XiaNH-072X-824 was also carried out by FISH (Additional file 1: Figure S4a–h). Repeat sequence *Ms*CR-1, *Ms*CR-2, *Ms*CR-3, *Ms*CR-4, and *Ms*CR-5 were physically mapped on pericentromeric regions of 30, 16, 7–8, 18, and 16 of the 32 chromosomes of tetraploid ssp. *falcata*, respectively (Additional file 1: Figure S4a–d). Double-target FISH revealed that *Ms*CR-3 overlapped with *Ms*CR-2, *Ms*CR-4, and *Ms*CR-5 on 4, 6, and 7 chromosomes, respectively. The subtelomeric sequence *Ms*TR-1 was co-localized with clone 65 on one end of 13 chromosomes and with clone 74 on one end of 17 chromosomes (Additional file 1: Figure S4e, f). Clone 65 produced more weak signals in the subtelomeric regions of two chromosomes than *Ms*TR-1 did (Additional file 1: Figure S4e), while clone 74 produced extra weak signals in the interstitial regions of two chromosomes compared with *Ms*TR-1 (Additional file 1: Figure S4f). E180, clone 68, and clone 87 were hybridized on 23–24, 29, and 32 chromosomes, respectively (Additional file 1: Figure S4g, h). Double-target FISH revealed that E180 was co-distributed with clone 68 and clone 87 on 20 and 24 chromosomes, respectively (Additional file 1: Figure S4g, h).

**Fig. 4:**
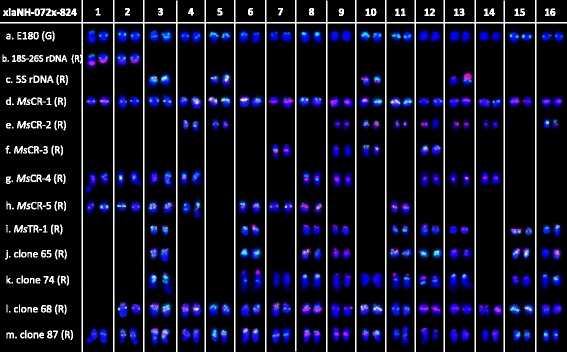
Localization of repeats on tetraploid *Medicago sativa* ssp. *falcata* XiaNH-072X-824 somatic chromosomes using **a** probe E180 (*green*) in combination with **b** 18S–26S rDNA, **c** 5S rDNA, **d** *Ms*CR-1, **e** *Ms*CR-2, **f** *Ms*CR-3, **g** *Ms*CR-4, **h** *Ms*CR-5, **i** *Ms*TR-1, **j** clone 65, **k** clone 74, **l** clone 68, and **m** clone 87

According to hybridization results between E180 and each repeat sequences, the chromosomal distribution of each sequence was allocated as follows (Fig. 4): 18S–26S rDNA: on chromosome 1 and 2; 5S rDNA: on chromosome 3, 5, 10, and 13; *Ms*CR-1: on all chromosomes; *Ms*CR-2: on chromosome 4, 5, 9–11, 13, 14, 16, and one of chromosome 12; *Ms*CR-3: on chromosome 7, 10, 12, and one of chromosome 9; *Ms*CR-4: on chromosome 1–4, 8, 9, 12, 13, and 14; *Ms*CR-5: on chromosome 1–3, 6–8, 11, and one of chromosome 4; *Ms*TR-1: on chromosome 3, 6, 8, 9, 11–13, 15, and one of chromosome 16; clone 65: on chromosome 3, 6, 8, 9, 11–13, 15, and one of chromosome 16; clone 74: on chromosome 3, 6, 7–9, 11–13, 15, and one of chromosome 16 and 10; clone 68: on chromosome 2–10, 12–16, and one of chromosome 11; and clone 87: all chromosomes.

**Fig. 5:**
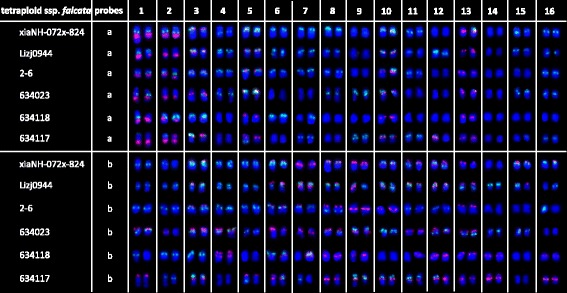
Karyotypes of six tetraploid *Medicago sativa* ssp. *falcata* accessions based on different FISH combinations. **a** Probed by E180 (*green*) combined with 18S–26S rDNA (*red*) and 5S rDNA (*red*). **b** Probed by E180 (*green*) combined with *Ms*TR-1 (*red*) and *Ms*CR-3 (*red*)

**Fig. 6:**
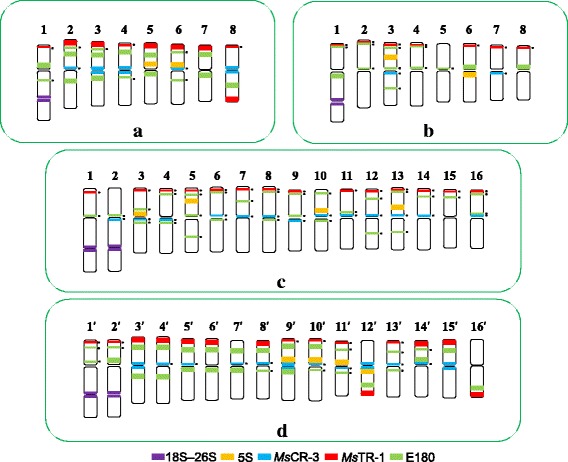
Idiogram of FISH-banded chromosomes of (**a**) *Medicago sativa* ssp. *caerulea*, (**b**) diploid *M. sativa* ssp. *falcata*, (**c**) tetraploid *M. sativa* ssp. *falcata*, and (**d**) *M. sativa* ssp. *sativa*. Idiogram of FISH-banded chromosomes of *M. sativa* ssp. *sativa* was summarized from four accessions [33]. Chromosomes of tetraploid ssp. *sativa* are marked with “′” on chromosome numbers. A small black dot next to the FISH signal indicates that the signal is polymorphic across accessions

The same two FISH cocktails with ssp. *caerulea* were applied to tetraploid ssp. *falcata* accession XiaNH-072X-824 and five other accessions (PI 634023, PI 634118, PI 634117, Lizj0944, and 2–6) to develop a standard molecular karyotype (Additional file 1: Figure S5). Marked variability was detected among the six tetraploid ssp. *falcata* accessions (Fig. 5). Thus only polymorphism schematic diagram was built in Fig. 6 (c).

### Comparative cytogenetic analysis between diploid and tetraploid subspecies

#### *M. sativa* ssp. *caerulea* and ssp. *sativa*

**Fig. 7:**
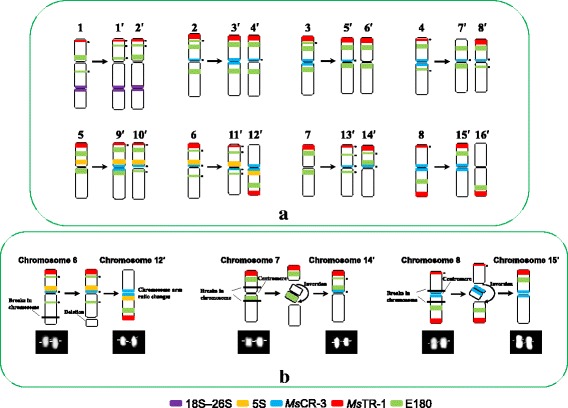
**a** Reconstructing of chromosome-doubling process of diploid ssp. *caerulea*. Chromosomes of tetraploid ssp. *sativa* are marked with “′” on chromosome numbers. **b** Putative chromosome changes during polyploidization process. A *small black dot* next to the FISH signal indicates that the signal is polymorphic across accessions

The comparative results of chromosome distribution of each repeat sequence (Table 2) showed that signals of all probes had similar chromosomal locations between ssp. *caerulea* (PI 464715) and ssp. *sativa* (Zhongmu No. 1). Moreover, the signal numbers of each probe were nearly twice between ssp. *caerulea* (PI 464715) and ssp. *sativa* (Zhongmu No. 1). Cocktail FISH results revealed the chromosome distributions of repeat sequences were highly conserved in four ssp. *caerulea* accessions and four ssp. *sativa* accessions, respectively (Fig. 6(a) and (d)). The high similar FISH patterns between ssp. *caerulea* and ssp. *sativa* facilitated the recognition of homoeologous chromosomes. Furthermore, autopolyplidization from ssp. *caerulea* to ssp. *sativa* was tentatively reconstructed. The reconstructed results showed though collinearity was well maintained in most chromosomes between diploid and tetraploid by FISH patterns, significant and stable variations were also detected in a few chromosomes (Fig. 7 (a)). Compared with chromosome 3 of ssp. *caerulea*, chromosome 6′ of ssp. *sativa* was missing the *Ms*CR-3 signal at pericentromeric region. Compared with chromosome 8 of ssp. *caerulea*, chromosome 16′ of ssp. *sativa* was missing the *Ms*CR-3 signal at pericentromeric region. Compared with chromosome 5 of ssp. *caerulea*, chromosome 9′ of ssp. *sativa* had an extra *Ms*CR-3 signal at pericentromeric region. Compared with chromosome 6 of ssp. *caerulea*, chromosome 12′ of ssp. *sativa* had 5S and *Ms*TR-1 repeat signals on long arm instead of short arm. Compared with chromosome 7 of ssp. *caerulea*, chromosome 14′ of ssp. *sativa* had one of E180 signals near the centromere of short arm instead of long arm. Compared with chromosome 8 of ssp. *caerulea*, chromosome 15′ of ssp. *sativa* had E180 signals on short arm instead of long arm. Combining all chromosome changes, chromosome deletion was speculated to occur on the long arm of chromosome 12′ of ssp. *sativa* during evolution. And pericentric inversions were speculated to occur in chromosome 14′ and chromosome 15′ of ssp. *sativa* during evolution. Putative chromosome changes were presented on Fig. 7 (b). Furthermore, significant variations between four groups of homologous chromosomes of tetraploid alfalfa were also detected (Fig. 7 (a)). Compared with homologous chromosome 8′, chromosome 7′ was missing the *Ms*TR-1 signals at subtelomeric region of short arm. Compared with homologous chromosome 11′, chromosome 12′ had 5 s signal on short arm instead of long arm. Compared with homologous chromosome 13′, chromosome 14′ had an extra E180 signals at the pericentromeric region of short arm. Compared with homologous chromosome 15′, chromosome 16′ was missing *Ms*CR-3 signal at pericentromeric region and had signals of *Ms*TR-1and E180 repeats on long arm instead of short arm.

**Table 2:** The comparison of chromosome distributions of each repeat sequence among four subspecies. Chromosome distributions of each repeat sequence in *Medicago sativa* ssp. *sativa* were summarized from Yu et al. [33]

Probes	ssp. *caerulea* (PI 464715)	ssp. *sativa* (Zhongmu No. 1)	Diploid ssp. *falcata* (PI 631808)	Tetraploid ssp. *falcata* (XiaNH-072X-824)
18S–26S	1 (secondary constriction)	2 (secondary constriction)	1 (secondary constriction)	2 (secondary constriction)
5S	2 (near centromeric)	4 (near centromeric)	2 (near centromeric and interstitial region)	4 (near centromeric and interstitial region)
*Ms*CR-1	14 (pericentromeric)	30–32 (pericentromeric)	16 (pericentromeric)	30–32 (pericentromeric)
*Ms*CR-2	8 (pericentromeric)	15 (pericentromeric)	10 (pericentromeric)	16–17 (pericentromeric)
*Ms*CR-3	6 (pericentromeric)	16 (pericentromeric)	0–1 (pericentromeric)	7–8 (pericentromeric)
*Ms*CR-4	10 (pericentromeric)	17 (pericentromeric)	9 (pericentromeric)	18 (pericentromeric)
*Ms*CR-5	9 (pericentromeric)	19 (pericentromeric)	8 (pericentromeric)	16–17 (pericentromeric)
*Ms*TR-1	12–13 (subtelomeric)	24–26 (subtelomeric)	1 (subtelomeric)	17 (subtelomeric)
clone 65	12–13 (subtelomeric)	26 (subtelomeric)	1 (subtelomeric)	13 (subtelomeric)
clone 74	12–13 (subtelomeric)	26 (subtelomeric)	1 (subtelomeric)	17 (subtelomeric)
E180	16 (multiple distribution)	30–32 (multiple distribution)	10–11 (mainly on pericentromeric)	23–24 (multiple distribution)
clone 68	15 (multiple distribution)	28 (multiple distribution)	16 (mainly on pericentromeric)	29 (multiple distribution)
clone 87	14 (multiple distribution)	32 (multiple distribution)	16 (mainly on pericentromeric)	32 (multiple distribution)

#### Diploid ssp. *falcata* and tetraploid ssp. *falcata*

The comparative results of chromosome distributions of each repeat sequence (Table 2) showed that signals of ten probes had similar chromosomal locations between diploid ssp. *falcata* (PI 631808) and tetraploid ssp. *falcata* (XiaNH-072X-824). However, signal distributions of E180, clone 68, and clone 87 probes were more abundant in tetraploid ssp. *falcata* than in diploid ssp. *falcata*. The signal numbers of *Ms*CR-1, *Ms*CR-2, *Ms*CR-4, *Ms*CR-5, E180, clone 68, and clone 87 probes were nearly twice between diploid ssp. *falcata* (PI 631808) and tetraploid ssp. *falcata* (XiaNH-072X-824). However, the signals of *Ms*CR-3, *Ms*TR-1, clone 65, and clone 74 probes was only located on one chromosome in diploid ssp. *falcata* rather than on many chromosomes in tetraploid ssp. *falcata*. Moreover, the cocktail FISH results revealed highly variable karyotypes across different tetraploid ssp. *falcata* accessions. Thus, chromosome collinearity analysis between diploid and tetraploid ssp. *falcata* could not be conducted as did between ssp. *caerulea* and ssp. *sativa*.

## Discussion

### Genomic differentiation of *M. sativa* ssp. *caerulea* and diploid ssp. *falcata*

*Medicago sativa ssp. caerulea* and diploid ssp. *falcata* are sympatrically distributed, with naturally occurring hybrids recorded between them [13, 14, 36]. The genetic affinity of the two species has been demonstrated by cytological research [15]. In addition, chromosomal differentiation between *M. sativa* ssp. *caerulea* and diploid ssp. *falcata* has been well described by analyses of both C- and N-banded chromosomes [20, 21]. C- and N-banding has revealed that chromosomes of diploid ssp. *falcata* possess only centromeric bands. In contrast, all chromosomes of ssp. *caerulea* have a centromeric band and a telomeric band in the short arm; in addition, most of the chromosomes of this subspecies have interstitial bands in the short arm, with a few chromosomes featuring prominent interstitial bands in the long arm.

C- and N-bands reflect constitutive heterochromatic DNA in chromosomes [23]. Our molecular cytogenetic analysis revealed the heterogeneous nature of the constitutive heterochromatin among centromeric, interstitial, and subtelomeric regions. In both ssp. *caerulea* and diploid ssp. *falcata*, centromeric bands were revealed to be a heterogeneous mix of *Ms*CR-1, *Ms*CR-2, *Ms*CR-3, *Ms*CR-4, *Ms*CR-5, clone 68, clone 87, and E180 sequences, along with a few 5S rDNA sites. The interstitial bands comprised E180 sequences along with 18S–26S rDNA and 5S rDNA sites, and the subtelomeric bands were represented by *Ms*TR-1, clone 68, clone 87, and E180.

Chromosomal differences between ssp. *caerulea* and diploid ssp. *falcata* as revealed by FISH were similar to those uncovered by C- or N-banding. The repetitive sequences were physically mapped onto centromeric, subtelomeric, or interstitial regions in ssp. *caerulea*, whereas the mapped sequences were mainly on centromeric regions in diploid ssp. *falcata*. Furthermore, conspicuous differences in distribution patterns were observed between ssp. *caerulea* and diploid ssp. *falcata*, even though the repetitive sequences detected in centromeric regions of both species displayed similar levels of heterogeneity. Unlike ssp. *caerulea*, more than half of the chromosomes of ssp. *falcata* contained E180 sequences in centromeric regions. In addition, centromeric sequences of *Ms*CR-3 were detected on one or no chromosomes of diploid ssp. *falcata*, whereas they were found on 3–5 pairs of chromosomes in ssp. *caerulea*.

Genetic differentiation between ssp. *caerulea* and diploid ssp. *falcata* has been previously revealed by nuclear markers [37–39]. Moreover, relationships uncovered among diploid members of the *M. sativa* species complex based on chloroplast DNA sequence analysis supports the recognition of ssp. *caerulea* and diploid ssp. *falcata* as distinct taxa [40]. Our study has revealed distinct genomic differentiation between ssp. *caerulea* and diploid ssp. *falcata* and supports their taxonomic differentiation at the chromosome level.

### Chromosome evolution after polyploidization of diploid ssp. *caerulea*

Violet flowered diploid ssp. *caerulea* is postulated to have given rise to tetraploid ssp. *sativa* (alfalfa) [13, 16, 17]. The identical C-banding patterns of tetraploid alfalfa and ssp. *caerulea* support tetraploid alfalfa as an autotetraploid derived from diploid ssp. *caerulea* [21, 23]. Sequencing of chloroplast DNA has demonstrated that the two taxa have very closely related chloroplast haplotypes, with most individuals sharing the same haplotype, and are thus undifferentiated genetically for this characteristic. Similar to the C-banding analysis, chloroplast data supports a simple autopolyploid origin for ssp. *sativa* from diploid ssp. *caerulea* [19]. In our study, a putative chromosome doubling process from diploid ssp. *caerulea* to tetraploid alfalfa was reconstructed according to similar FISH patterns. The results strongly supported the simple autotetraploid origin of ssp. *sativa* from diploid ssp. *caerulea*.

It is generally believed that polyploid plants may have unstable genomes in a long term due to a genome-wide gene redundancy [41]. Ma and Gustafson [42] summarized the evolution of an allopolyploid species is a process of both cytological and genetic diploidization. Rapid genomic rearrangement such as chromosome insertion, chromosome deletion, and chromosome rearrangement, which would lead to diploidization of genome structure, has been investigated in some allopolyploid plant species [41, 43]. However, the occurrence of similar changes remains to be studied in detail during the generation of autopolyploids [44]. The limited data available so far imply that autopolyploids experience less genome restructuring than allopolyploids [44]. In our study, putative genome changes were discovered after polyploidization of diploid ssp. *caerulea*. Elimination of repetitive DNA was detected in pericentromeric regions of chromosome 6′ and chromosome 16′ of tetraploid ssp. *sativa*. Increase of repetitive DNA was detected in pericentromeric regions of chromosome 9′ of tetraploid ssp. *sativa*. Chromosome deletion was postulated to occur in the long arm of chromosome 12′ of tetraploid ssp. *sativa*. Pericentric inversions were postulated to occur in chromosome 14′ and chromosome 15′ of tetraploid ssp. *sativa*. Furthermore, significant diversification was recognized in four groups of homologous chromosome of tetraploid ssp. *sativa* including chromosome 7′ and chromosome 8′, chromosome 11′ and chromosome 12′, chromosome 13′ and chromosome 14′ and chromosome 15′ and chromosome 16′. Thus, we concluded that autotetraploid alfalfa had undergone a partial diploidization by the progressive accumulation of chromosome structural rearrangements during evolution. A previous study of pachytene karyotype reported that at least four groups of the tetraploid chromosomes appear sufficiently alike to be able to form quadrivalents in ssp. *sativa*, and three of these were seen to form quadrivalents at pachytene [45]. Subsequently, Armstrong summarized the quadrivalent frequency at pachytene ranged from 0.89 to 2.93 in tetraploid ssp. *sativa* [46]. It was considerably below theoretical expectations “5.34 quadrivalents per cell” for an autotetraploid [46]. This chromosome behavior in meiotic has confused the origin of tetraploid ssp. *sativa*. Partial diploidization of homologous chromosome groups in tetraploid *M. sativa* found in our study should be an explanation for the low quadrivalent frequencies at meiotic.

### Phylogenetic relationships between diploid and tetraploid forms of ssp. *falcata*

Diploid and tetraploid forms of ssp. *falcata* have been traditionally treated as a single species, *M. sativa* ssp. *falcata*. Diploid ssp. *falcata* and tetraploid ssp. *falcata* have been recognized as diploid and tetraploid cytotypes on the basis of chromosome counting and morphology. Diploid ssp. *falcata* is hypothesized to be the ancestor of autoploid tetraploid ssp. *falcata* [13, 14]. As revealed by C- and N-banding, centromeric bands are a distinct feature of the chromosomes of diploid ssp. *falcata* [20, 21]. Because of this assumption of autopolyploid origin, the C-banding pattern of tetraploid ssp. *falcata* was expected to be similar to that of diploid ssp. *falcata*. Thus, the results of a preliminary study of six accessions of tetraploid ssp. *falcata* were surprising. Most of the plants possessed chromosomes that had C-bands in addition to normal centromeric bands [12]. Highly variable C-banding patterns were detected in these accessions. The accession containing the fewest number of additional bands had four pairs of chromosomes with an extra telomeric band on their short arms, whereas the remaining chromosomes had only centromeric bands. At the other extreme, two accessions had multiple bands on each chromosome, similar to doubled-diploid ssp. *caerulea*. Even though the studied accessions had yellow flowers with sickle-shaped pods, the accessions were speculated to be the product of hybridization with ssp. *sativa* [12]. Similarly, the six tetraploid ssp. *falcata* accessions used in our study—three acquired from the NPGS USDA germplasm bank and three collected in situ in China on the basis of morphological identification—also showed highly variable molecular karyotypes. We found that hybridization sites of *Ms*TR-1 and E180, which frequently produce subtelomeric and interstitial bands, respectively, in C-banding analyses, were highly variable across different individuals of tetraploid ssp. *falcata*. Along with the results of C-banding analysis [12], our results suggested the actual genomic characteristics of tetraploid ssp. *falcata.*

In an earlier analysis of chloroplast DNA, morphologically identical diploid and tetraploid cytotypes of ssp. *falcata* were found to possess very different chloroplast haplotypes. The most common haplotype of tetraploid ssp. *falcata* was shared with *M. prostrata* rather than diploid ssp. *falcata*, suggesting past introgression from *M. prostrata* into the polyploid. The evolutionary trajectory of ssp. *falcata* does not appear to have involved a simple autopolyploid origin as seen in ssp. *sativa* [19]. A high variability in the number of chromosomes with multiple E180 sites, which are frequently lacking in diploid ssp. *falcata*, was uncovered in our study. Although information on the chromosomal distribution of E180 in *M. prostrata* is not available, multiple E180 hybridization signals have been detected in species of section *Medicago*, such as *M. glutinosa*, *M. hemicycla*, and *M. polychroa* [47]. This finding suggests that the variable chromosomes in tetraploid ssp. *falcata* could have been introduced from *M. prostrata*. Our data indicate that the origin of tetraploid ssp. *falcata* from diploid ssp. *falcata* is far from simple. Elucidation of the evolutionary history of ssp. *falcata* will require a large amount of additional data.

## Conclusions

The comparative cytogenetic results provide reliable evidence that diploid subspecies *caerulea* is the direct progenitor of tetraploid subspecies *sativa*. And autotetraploid ssp. *sativa* has been suggested to undergo a partial diploidization by the progressive accumulation of chromosome structural rearrangements during evolution. However, the tetraploid subspecies *falcata* is far from a simple autopolyploid from diploid subspecies *falcata* although no obvious morphological change was observed between these two subspecies.

## Additional file

Additional file 1: Figure S1-S6. (PPTX 2091 kb)

## Abbreviations

FISH: Fluorescence in situ hybridization
NPGS: National Plant Germplasm System
USDA: United States Department of Agriculture
