# Supplementary material for: Karyotypic evolution of the Medicago complex: sativa-caerulea-falcata inferred from comparative cytogenetic analysis
Source: BMC Evol Biol. 2017 Apr 21;17:104. doi: 10.1186/s12862-017-0951-x (PMC5399346; doi:10.1186/s12862-017-0951-x)
Supplement: Additional file 1: — Figure S1-S6. (PPTX 2091 kb) [file 12862_2017_951_MOESM1_ESM.pptx]

## Slide 1
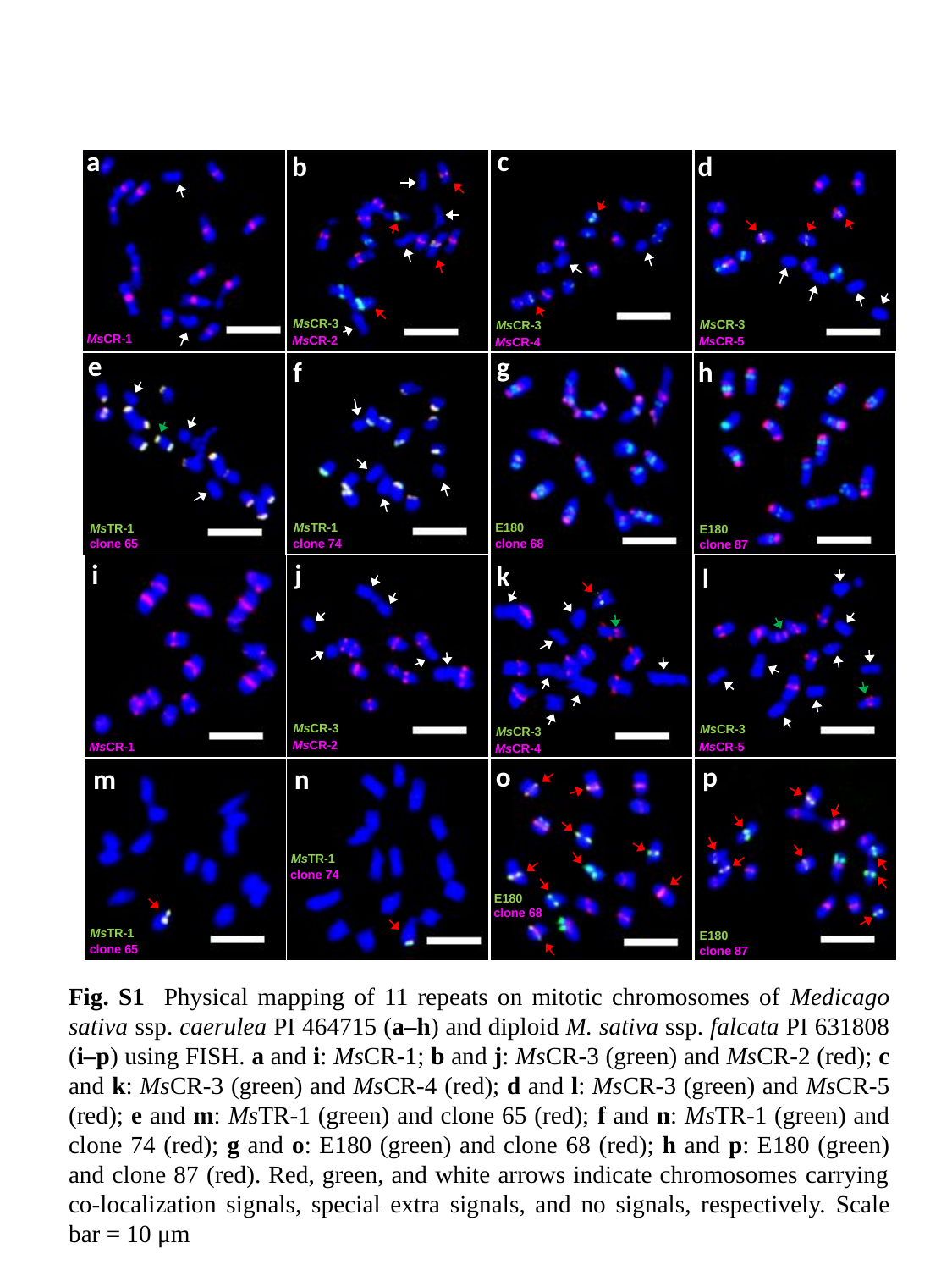

c
a
b
d
e
g
f
h
i
j
k
l
p
o
m
n
MsCR-3
MsCR-3
MsCR-3
MsCR-1
MsCR-2
MsCR-5
MsCR-4
MsTR-1
E180
MsTR-1
E180
clone 74
clone 68
clone 65
clone 87
MsCR-3
MsCR-3
MsCR-3
MsCR-2
MsCR-5
MsCR-1
MsCR-4
MsTR-1
clone 74
E180
clone 68
MsTR-1
E180
clone 65
clone 87
Fig. S1 Physical mapping of 11 repeats on mitotic chromosomes of Medicago sativa ssp. caerulea PI 464715 (a–h) and diploid M. sativa ssp. falcata PI 631808 (i–p) using FISH. a and i: MsCR-1; b and j: MsCR-3 (green) and MsCR-2 (red); c and k: MsCR-3 (green) and MsCR-4 (red); d and l: MsCR-3 (green) and MsCR-5 (red); e and m: MsTR-1 (green) and clone 65 (red); f and n: MsTR-1 (green) and clone 74 (red); g and o: E180 (green) and clone 68 (red); h and p: E180 (green) and clone 87 (red). Red, green, and white arrows indicate chromosomes carrying co-localization signals, special extra signals, and no signals, respectively. Scale bar = 10 μm

## Slide 2
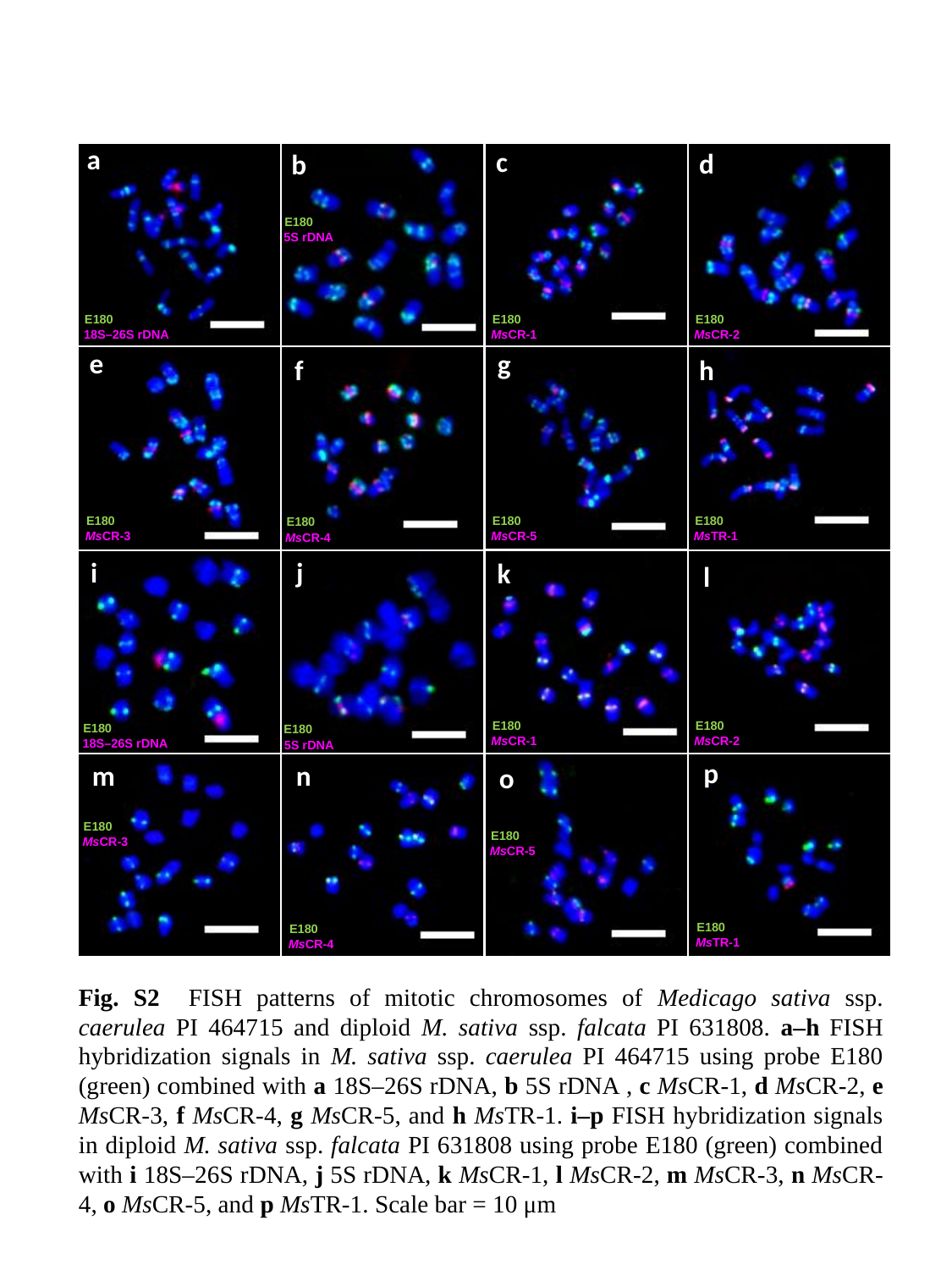

a
c
d
b
e
g
f
h
i
j
k
l
p
m
n
o
E180
5S rDNA
E180
E180
E180
18S–26S rDNA
 MsCR-1
 MsCR-2
E180
E180
E180
E180
 MsCR-3
 MsCR-5
 MsTR-1
 MsCR-4
E180
E180
E180
E180
 MsCR-1
 MsCR-2
18S–26S rDNA
5S rDNA
E180
E180
 MsCR-3
 MsCR-5
E180
E180
 MsTR-1
 MsCR-4
Fig. S2 FISH patterns of mitotic chromosomes of Medicago sativa ssp. caerulea PI 464715 and diploid M. sativa ssp. falcata PI 631808. a–h FISH hybridization signals in M. sativa ssp. caerulea PI 464715 using probe E180 (green) combined with a 18S–26S rDNA, b 5S rDNA , c MsCR-1, d MsCR-2, e MsCR-3, f MsCR-4, g MsCR-5, and h MsTR-1. i–p FISH hybridization signals in diploid M. sativa ssp. falcata PI 631808 using probe E180 (green) combined with i 18S–26S rDNA, j 5S rDNA, k MsCR-1, l MsCR-2, m MsCR-3, n MsCR-4, o MsCR-5, and p MsTR-1. Scale bar = 10 μm

## Slide 3
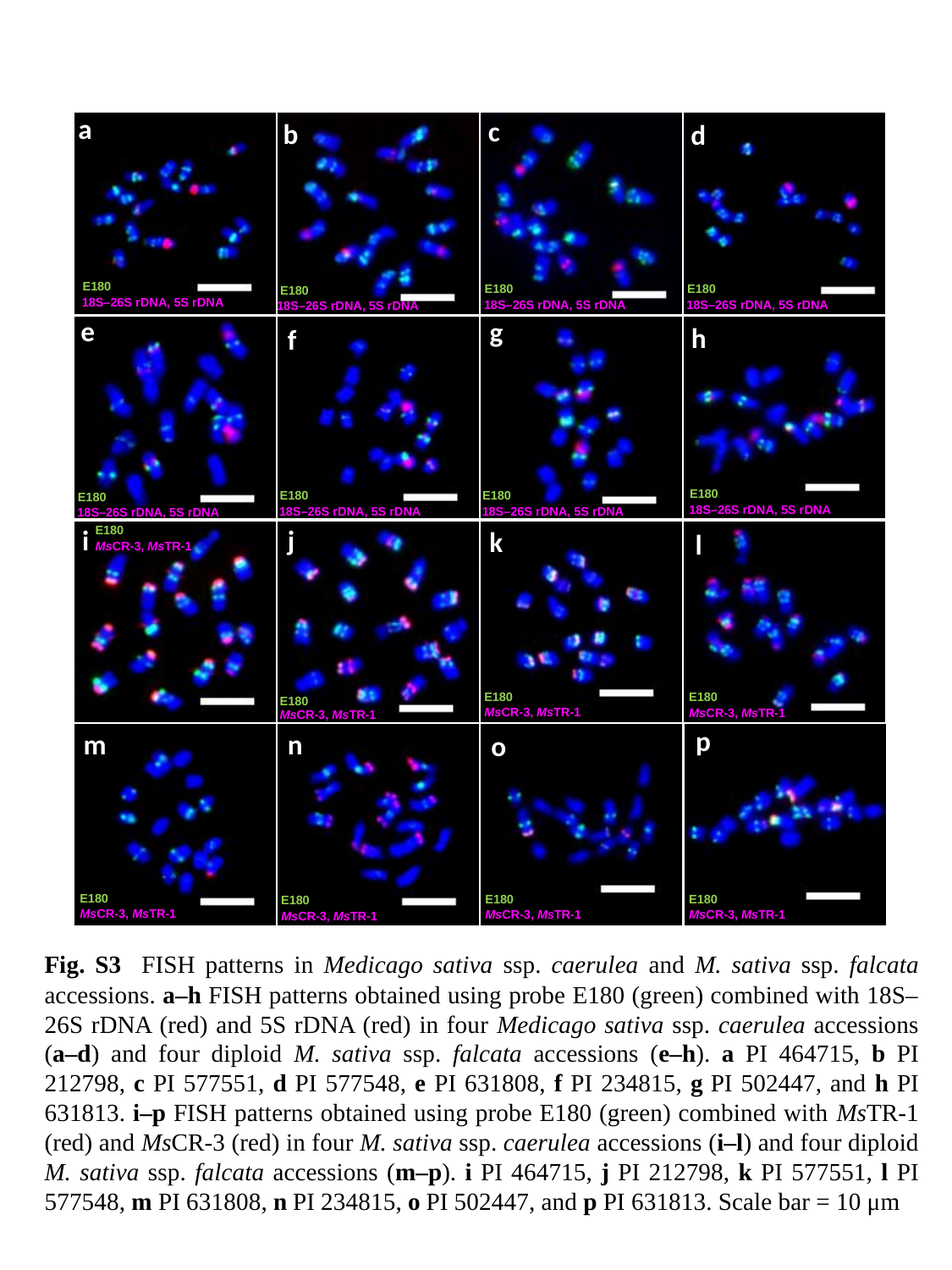

a
c
b
d
e
g
h
f
i
j
k
l
p
m
n
o
E180
E180
E180
E180
 18S–26S rDNA, 5S rDNA
 18S–26S rDNA, 5S rDNA
 18S–26S rDNA, 5S rDNA
 18S–26S rDNA, 5S rDNA
E180
E180
E180
E180
 18S–26S rDNA, 5S rDNA
 18S–26S rDNA, 5S rDNA
 18S–26S rDNA, 5S rDNA
 18S–26S rDNA, 5S rDNA
E180
MsCR-3, MsTR-1
E180
E180
E180
MsCR-3, MsTR-1
MsCR-3, MsTR-1
MsCR-3, MsTR-1
E180
E180
E180
E180
MsCR-3, MsTR-1
MsCR-3, MsTR-1
MsCR-3, MsTR-1
MsCR-3, MsTR-1
Fig. S3 FISH patterns in Medicago sativa ssp. caerulea and M. sativa ssp. falcata accessions. a–h FISH patterns obtained using probe E180 (green) combined with 18S–26S rDNA (red) and 5S rDNA (red) in four Medicago sativa ssp. caerulea accessions (a–d) and four diploid M. sativa ssp. falcata accessions (e–h). a PI 464715, b PI 212798, c PI 577551, d PI 577548, e PI 631808, f PI 234815, g PI 502447, and h PI 631813. i–p FISH patterns obtained using probe E180 (green) combined with MsTR-1 (red) and MsCR-3 (red) in four M. sativa ssp. caerulea accessions (i–l) and four diploid M. sativa ssp. falcata accessions (m–p). i PI 464715, j PI 212798, k PI 577551, l PI 577548, m PI 631808, n PI 234815, o PI 502447, and p PI 631813. Scale bar = 10 μm

## Slide 4
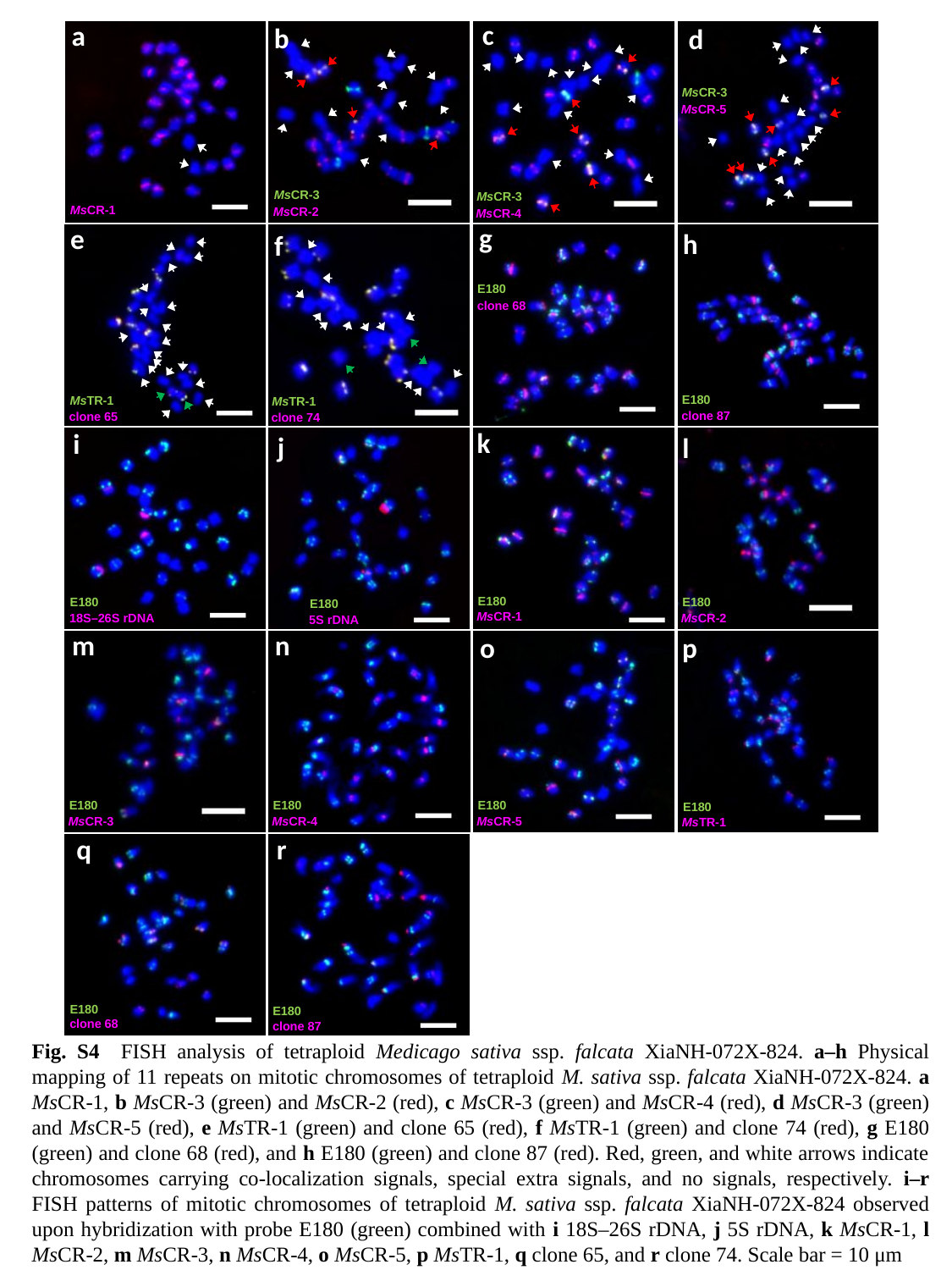

c
a
b
d
MsCR-3
MsCR-5
MsCR-3
MsCR-3
MsCR-1
MsCR-2
MsCR-4
g
e
h
f
E180
clone 68
E180
MsTR-1
MsTR-1
clone 87
clone 65
clone 74
k
i
j
l
E180
E180
E180
E180
 MsCR-1
18S–26S rDNA
 MsCR-2
5S rDNA
n
m
o
p
E180
E180
E180
E180
 MsCR-3
 MsCR-4
 MsCR-5
 MsTR-1
q
r
E180
E180
clone 68
clone 87
Fig. S4 FISH analysis of tetraploid Medicago sativa ssp. falcata XiaNH-072X-824. a–h Physical mapping of 11 repeats on mitotic chromosomes of tetraploid M. sativa ssp. falcata XiaNH-072X-824. a MsCR-1, b MsCR-3 (green) and MsCR-2 (red), c MsCR-3 (green) and MsCR-4 (red), d MsCR-3 (green) and MsCR-5 (red), e MsTR-1 (green) and clone 65 (red), f MsTR-1 (green) and clone 74 (red), g E180 (green) and clone 68 (red), and h E180 (green) and clone 87 (red). Red, green, and white arrows indicate chromosomes carrying co-localization signals, special extra signals, and no signals, respectively. i–r FISH patterns of mitotic chromosomes of tetraploid M. sativa ssp. falcata XiaNH-072X-824 observed upon hybridization with probe E180 (green) combined with i 18S–26S rDNA, j 5S rDNA, k MsCR-1, l MsCR-2, m MsCR-3, n MsCR-4, o MsCR-5, p MsTR-1, q clone 65, and r clone 74. Scale bar = 10 μm

## Slide 5
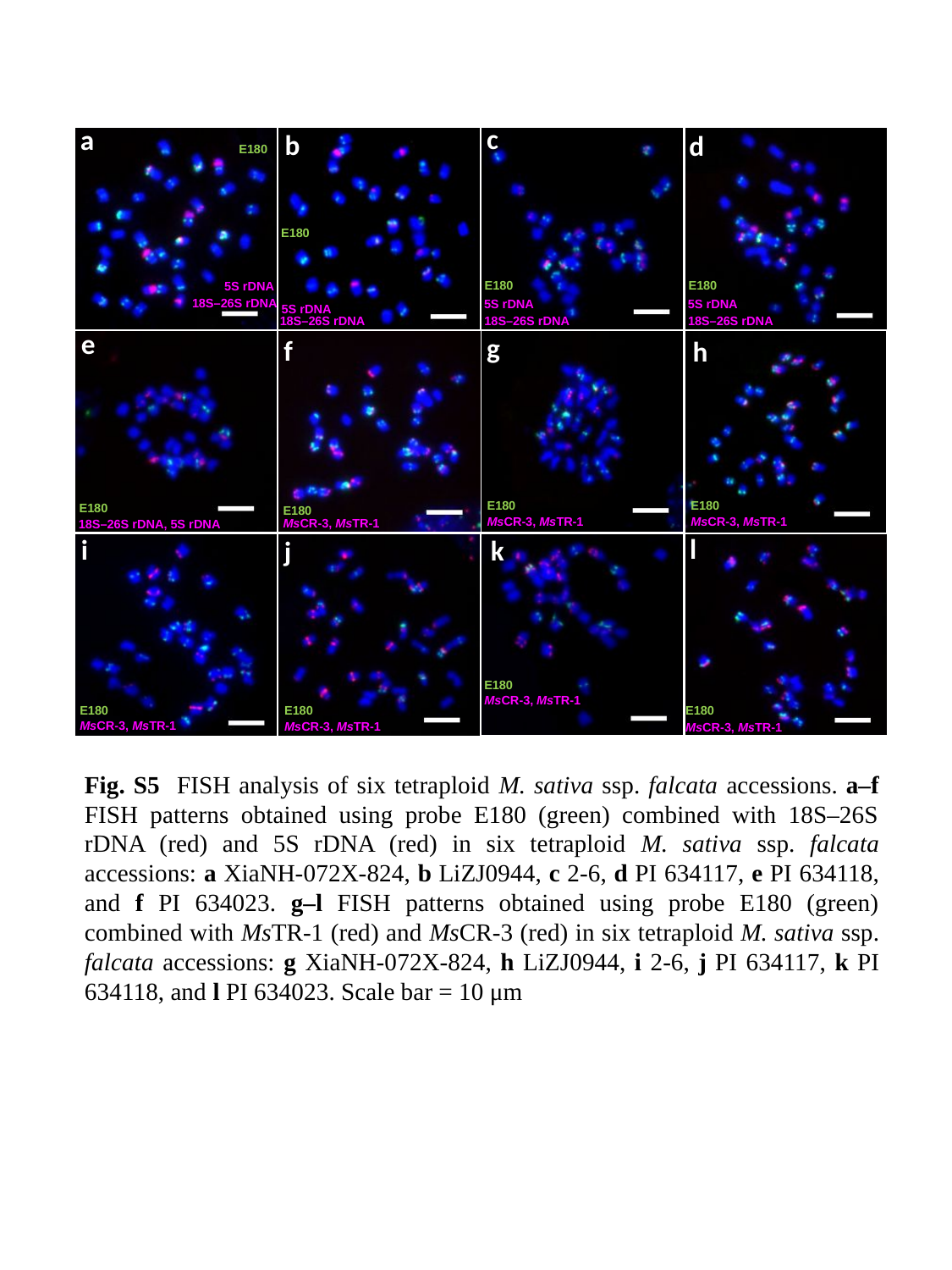

c
a
b
d
e
g
f
h
l
i
k
j
E180
E180
E180
E180
5S rDNA
 18S–26S rDNA
5S rDNA
5S rDNA
5S rDNA
 18S–26S rDNA
 18S–26S rDNA
 18S–26S rDNA
E180
E180
E180
E180
MsCR-3, MsTR-1
MsCR-3, MsTR-1
MsCR-3, MsTR-1
 18S–26S rDNA, 5S rDNA
E180
MsCR-3, MsTR-1
E180
E180
E180
MsCR-3, MsTR-1
MsCR-3, MsTR-1
MsCR-3, MsTR-1
Fig. S5 FISH analysis of six tetraploid M. sativa ssp. falcata accessions. a–f FISH patterns obtained using probe E180 (green) combined with 18S–26S rDNA (red) and 5S rDNA (red) in six tetraploid M. sativa ssp. falcata accessions: a XiaNH-072X-824, b LiZJ0944, c 2-6, d PI 634117, e PI 634118, and f PI 634023. g–l FISH patterns obtained using probe E180 (green) combined with MsTR-1 (red) and MsCR-3 (red) in six tetraploid M. sativa ssp. falcata accessions: g XiaNH-072X-824, h LiZJ0944, i 2-6, j PI 634117, k PI 634118, and l PI 634023. Scale bar = 10 μm

## Slide 6
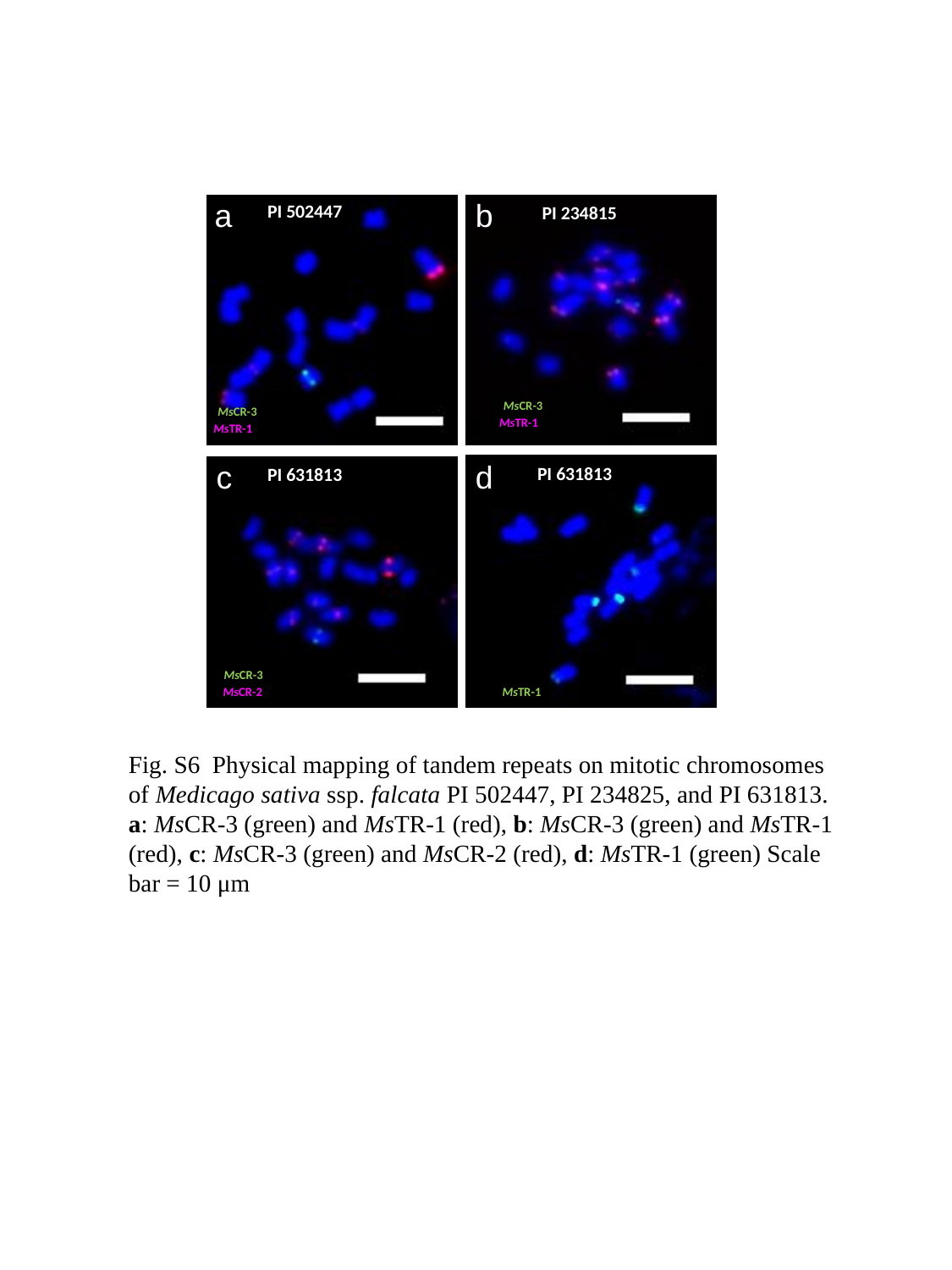

a
b
PI 502447
PI 234815
MsCR-3
MsCR-3
MsTR-1
MsTR-1
c
d
PI 631813
PI 631813
MsCR-3
MsCR-2
MsTR-1
Fig. S6 Physical mapping of tandem repeats on mitotic chromosomes of Medicago sativa ssp. falcata PI 502447, PI 234825, and PI 631813. a: MsCR-3 (green) and MsTR-1 (red), b: MsCR-3 (green) and MsTR-1 (red), c: MsCR-3 (green) and MsCR-2 (red), d: MsTR-1 (green) Scale bar = 10 μm
